# Naturally Occurring Follicle-Stimulating Hormone Glycosylation Variants

**DOI:** 10.4172/2153-0637.1000e117

**Published:** 2014

**Authors:** John S Davis, T Rajendra Kumar, Jeffrey V May, George R Bousfield

**Affiliations:** 1 VA Nebraska-Western Iowa Health Care System and Olson Center for Women’s Health, University of Nebraska Medical Center, Omaha, Nebraska, USA; 2 Department of Molecular and Integrative Physiology, University of Kansas Medical Center, Kansas City, Kansas, USA; 3 Department of Biological Sciences, Wichita State University, Wichita, Kansas, USA

Follicle-stimulating hormone (FSH) is a member of the glycoprotein hormone family, which is a subfamily of the cystine knot growth factor superfamily [1,2]. The glycoprotein hormones are composed of heterodimeric glycoprotein subunits, a common α-subunit, and a hormone-specific β-subunit. While the α-subunit primary structure is identical for all glycoprotein hormones within the same species, the oligosaccharide populations differ in a hormone-specific manner [3–6]. Characterizing the oligosaccharides released from an α-subunit preparation can identify the hormone from which the subunit was derived [7]. There are 3 to 4 β-subunits in vertebrates, which combine with α-subunit to create either FSH, luteinizing hormone (LH), thyroid-stimulating hormone (TSH), or in primates and equids, chorionic gonadotropin (CG) [8]. As both glycoprotein hormone subunits are cystine knot proteins [9–11] the protein backbone is folded into a series of three loops, two relatively rigid hairpin loops on one side of the knot, designated L1 and L3, and a single, flexible loop on the other side [12], designated L2. Oligosaccharides are attached to all 3 loops in a subunit-specific pattern (Figure 1). FSH subunits possess two potential N-glycosylation sites on each subunit and all four are of the Asn-Xaa-Thr type, which exhibit very efficient carbohydrate attachment [13]. Indeed, the α-subunit is always glycosylated at both sites in all known glycoprotein hormones. Because FSH α and β subunits co-migrate during electrophoresis, it is difficult to detect missing N-glycans in this hormone. FSHβ-specific Western blots have revealed partial glycosylation in equine FSHβ, human FSHβ (hFSH β), rhesus FSH β, and Japanese macaque FSHβ [14–16]. During the past few years, we have studied partially glycosylated hFSH isolated from pituitary extracts, postmenopausal urine, and conditioned tissue culture medium containing recombinant hFSH. Each glycosylation site in hFSH is decorated with a population of N-glycans. When total glycans are removed from reduced, carboxy-methylated FSH subunits, 39–130 glycans are found in mass spectra. We have data from only one glycosylation site, αAsn^52^, which possessed 29 neutral core ions, and when decorated with various patterns of sialic acid grew to 109 unique glycan structures. Micro heterogeneity can affect electrophoretic mobility, for example, placental hCGα with hybrid and biantennary glycans migrated faster than pituitary hFSHα, with triantennary, biantennary and tetraantennary glycans, which complicated sorting out the hFSH variants that resulted from loss of one or more N-glycans [17].

We have identified four hFSH variants, based on loss of one or more FSHβ N-glycans (Figure 2). We first encountered these on the basis of FSHβ-specific Western blot analysis. Recall that the α-subunit always possesses both N-glycans. FSHβ possessing both N-glycans migrates as a 24 KDa band, therefore, we designated this intact heterodimer as hFSH^24^. Two single-glycan variants provide 18 and 21 KDa bands, which represents the loss of Asn^7^ and Asn^24^ glycans, respectively. Peptide-N-glycanase F-de-glycosylated hFSH β migrates as a 15 KDa band and the corresponding heterodimer is designated as hFSH^15^. Expression of a recombinant hFSHβ subunit mutant that prevents glycosylation at both Asn^7^ and Asn^24^ glycosylation sites in transformed GH_3_ cells or in pituitaries of transgenic mice also produces a 15 KDa FSHβ band. Three of these variants, hFSH^18^, hFSH^21^, and hFSH^24^ are secreted. Most pituitary, urinary, and recombinant hFSH preparations that we have examined consist of two glycoforms, hFSH^24^ and hFSH^21^ in an 80:20 ratio [15,16].

Evaluation of hFSH in the pituitaries of adult women (ages 21 to 81) revealed a progressive loss of hFSH^21^ between ages 24 and 55, suggesting that the ratio of FSH^21^ to FSH^24^ deceases as a function of aging. In late reproductive age, there is a rise in circulating hFSH that begins about 6 years before the final menstrual period. This has been attributed to the reduced ability to stimulate steroidgenesis in the ovary, leading to a compensatory increase in FSH output by the pituitary that keeps circulating estrogen levels within the normal range until about 2 years before the final menstrual period [18]. Disrupted hormonal feedback from the ovary results in an increased molecular size of pituitary FSH in ovariectomized rhesus and rat females, as indicated by gel filtration chromatography, which is reversed by estrogen replacement therapy [19,20]. FSH is also regulated by the inhibins and it has been suggested that the increases in FSH during the peri-menopausal period are likely due to a reduction in ovarian follicle production of inhibin-B, because estradiol levels remained unchanged during this period. The role of inhibin in regulating FSH glycosylation has not been extensively investigated. Activin-A treatment of dispersed rat pituitary cells resulted in secretion of more acidic forms of FSH [21]. In the same report, estrogen treatment of these cells also resulted in the secretion of more acidic forms of FSH. However, subsequent studies in rats indicated that estrogen inhibited pituitary expression of α 2-3-sialyltransferase [22,23], suggesting that α2-6-sialyltransferase activity increased to compensate for the loss of one isoform. GnRH was reported to increase galactose content of LH glycans [24], indicating increased branching. GnRH stimulation of human subjects resulted in release of less acidic forms of hFSH into the serum [25,26] and secretion of less acidic FSH forms from dispersed rat pituitary cells, even in the presence of estrogen [21]. The mechanisms responsible for the increased formation of the fully glycosylated FSH^24^ that occurs during reproductive aging are not yet clear.

Isoform studies, which focus on the theoretical number of negatively charged sialic acid residues attached to FSH, generally report that less acidic FSH isoforms are more active in receptor-binding and *in vitro* steroidgenesis assays [27–31]. In contrast, acidic forms of FSH are more active *in vivo*, presumably because of longer survival in the circulation [27,32]. How do glycoforms lacking one or two β-subunit N-glycans fit into the isoform picture? Not well. Chromatofocusing of purified pituitary hFSH produced less acidic fractions consisting of hFSH^21^, followed by mixtures of hFSH^24^ and hFSH^21^, and all subsequent increasingly acidic fractions also consisted of both glycoforms instead of becoming largely, if not exclusively hFSH^24^ [15]. Analysis of a second set of hFSH isoforms separated by chromatofocusing revealed all but one fraction possessed both glycoforms [33]. Glycopeptide mass spectrometry of purified hFSH isoforms, comprised of mixtures of hFSH^21^ and hFSH^24^ derived from the second study, showed the glycan populations at αAsn^52^ and βAsn^24^ were virtually identical in all isoform fractions. Thus, it is quite difficult to reconcile FSH glycosylation macro-heterogeneity representing the four hFSH^24^, hFSH^21^, hFSH^18^, and hFSH^15^ glycoforms with micro-heterogeneity resulting from the 30 to over 100 glycans attached to as many as 4 Asn residues on the α and βFSH subunits. Modern methods of mass spectrometry have made it possible to compare two FSH glycan populations using as little as 10 μg samples of each preparation (the larger amounts of glycoprotein are dictated by the 80–139 glycans, not 4, that can be identified in these small samples) [34].

In order to establish the existence of FSH glycoforms, it is necessary to biochemically separate them so that they can be studied separately and the results compared. The residual FSH activity in LH preparations was captured by immuneaffinity chromatography and lacked hFSH^24^, but consisted of both hFSH^21^ and hFSH^18^ (we refer to such mixtures as hFSH^21/18^, the first superscript indicating the more abundant form). What captured our attention was the fact that this preparation was about 10-fold more active than highly purified hFSH^24/21^ and a hFSH^24^ hybrid prepared from FSHβ^24^ combined with hCGα [17]. Moreover, hFSH^21/18^ associated more rapidly with FSH receptors (FSHR) and occupied 2- to 3-fold more receptor sites than hFSH^24^.

Recent developments in the understanding of FSHR structure and function suggest that a reevaluation of the modulatory effects of FSH glycosylation on FSHR binding, receptor activation, and signaling. The FSHR, once considered a monomeric unit [35] with a mature receptor molecular weight of 74 kDa [36], is now recognized as at least a dimeric form [37–39] and there is biochemical evidence for higher order combinations of FSHRs [38,39]. In fact, ligand-binding studies suggested that the only functional FSHR form following SDS-PAGE and electro blotting to PVDF was a 200–240 kDa form [40,41]. The crystal structure of the complete FSHR extracellular domain (FSHR_ecd_) showed a trimeric structure with endoglycosidase F-deglycosylated FSH bound to each FSHR_ecd_ [42]. However, the location of the surviving α-subunit Asn^52^ GlcNAc residue suggested typical oligosaccharides attached to this position could make it impossible for more than one FSH to associate with this trimeric structure at the same time. While a certain amount of caution is in order because a FSHR high affinity site (FSHR_has_) dimeric model showed receptor dimerization via the extracellular domain in the crystal structure and provided evidence that such dimers could exist in solution [43]. However, this model was not supported a by subsequent study aimed at testing the dimerization mechanism [39]. Nevertheless, studies with intact FSH receptor showed that elimination of αAsn^52^ glycans in hFSH resulted in a 3-fold increase in receptor occupancy as compared to fully glycosylated recombinant hFSH [44]. Our studies indicate that hFSH^21/18^, which lacks one of the two FSHβ subunit N-glycans, also exhibits 2- to 3-fold higher saturation binding to the same FSHR preparation. This is intriguing since these glycans would not be expected to affect binding to the trimeric FSHR model, as they are oriented away from the center of the cluster. The hCGα: hFSHβ^24^ hybrid FSHR binding data support the αAsn^52^ model, as it exhibited reduced affinity and binding at saturation [17]. The three major glycans present at this site are hybrid type, possessing the complex lactosamine-type branch on the 3-position of the penta-saccharide core, and differ by the presence and linkage of a single mannose residue on the 6-postion. These oligosaccharides, consisting of 8–9 monosaccharide residues, reduce the number of FSHR sites that can be occupied simultaneously. In contrast, a single GlcNAc residue on each permits three FSH molecules to simultaneously bind to the proposed FSHR timer. Therefore, small oligosaccharides at this position should also permit higher receptor occupancy. The problem is that hFSH oligosaccharides are dominated by bi-, tri-, and tetra-antennary glycans [45–47]. However, mass spectrometry of FSHα Asn^56^ glycans, selectively released by peptide-N-glycanase F digestion, revealed several small, oligomannose glycans that may be compatible with simultaneous binding of more than one FSH to trimeric FSHR. This approach also revealed that 60% of total hFSH^21/18^ glycans were oligo mannose-type, although their location is not yet known and likely to critical. The good news is that αAsn^52^ glycans are the most accessible glycans in FSH. Dissociating FSH subunits followed by peptide-N-glycanase F digestion selectively removes this glycan, leaving all other glycans attached to partially deglycosylated FSH subunits [7,48,49]. The bad news is that the 10 μg sample size has to be increased to ~40 μg to provide enough glycan for nano-electrospray mass spectrometry analysis from a single site as compared with a total glycan population from an average of 3.8 sites (accommodating the presence of both hFSH^24^ and hFSH^21^). The reason is that hFSH glycoform preparations are difficult to prepare and existing techniques are quite inefficient. Nevertheless, the sacrifice of significant amounts of scarce hormone is certainly worthwhile to address an important question like do hFSH^21^ or hFSH^18^ preparations possess largely small αAsn^52^ glycans, enabling them to occupy more FSHR binding sites?

In the G protein coupled receptor (GPCR) field, including FSHR, biased signaling is coming under increasing scrutiny [50–52]. The realization that one GPCR can activate several signaling molecules to activate different pathways calls for the reinvestigation of previously confusing data. For example, both FSHR and LH/CGR primarily signal via Gαs leading to the activation of the cAMP/protein kinase A (PKA) pathway and subsequently leading to steroidgenesis [53–55]. Alternative pathways, such as phospholipase C/inositol triphosphate metabolism were first recognized over 25 years ago [56,57], however, most studies examining the actions of gonadotropin glycosylation variants remain fixed on the primary pathway. The concept of biased signaling predicts that the specificity of signal transduction depends on, at least in part, the structure of the ligand [reviewed in [50,51]]. In support of this idea, a partially deglycosylated LH variant [58] (eLHdg) was found to exhibit biased signaling through the FSHR [59]. While incapable of activating the cAMP/PKA pathway and eliciting steroidgenesis in granulosa cells, binding of eLHdg to FSHR recruited β-arrestins and activated ERK MAPK signaling via a cAMP-independent pathway. Another recent study showed that the oligosaccharide complexity of recombinant hFSH preparations differentially affected gene expression and steroidgenesis in human granulosa cells [60]. Our own studies with hFSH glycoforms have found evidence for biased signaling, albeit in different cell types.

The hFSH^21/18^ glycoforms were more active than hFSH^24^ in activating the cAMP/PKA pathway via Gαs in gonadal cells, while hFSH^24^ was more active in activating osteoclast differentiation via NFκB and MAPK signaling independent of Gαs-mediated cAMP/PKA signaling [61]. The obvious next step is to determine if this biased signaling by hFSH^24^ occurs in gonadal cells as well. Our group is actively pursuing this issue using both *in vitro* and *in vivo* genetic approaches.

The recently emerging concepts of FSHR working as dimers, trimers, or some other oligomer and biased signaling in response to altered FSH glycosylation open up new avenues for solving the more than 30 year old mystery of how full activation of FSHR and LHR require glycosylated FSH and LH preparations, despite the fact that receptor binding is exclusively a protein-protein interaction and the glycans appear to merely get in the way. These are exciting times for those studying gonadotropin glycosylation.

## Figures and Tables

**Figure 1:**
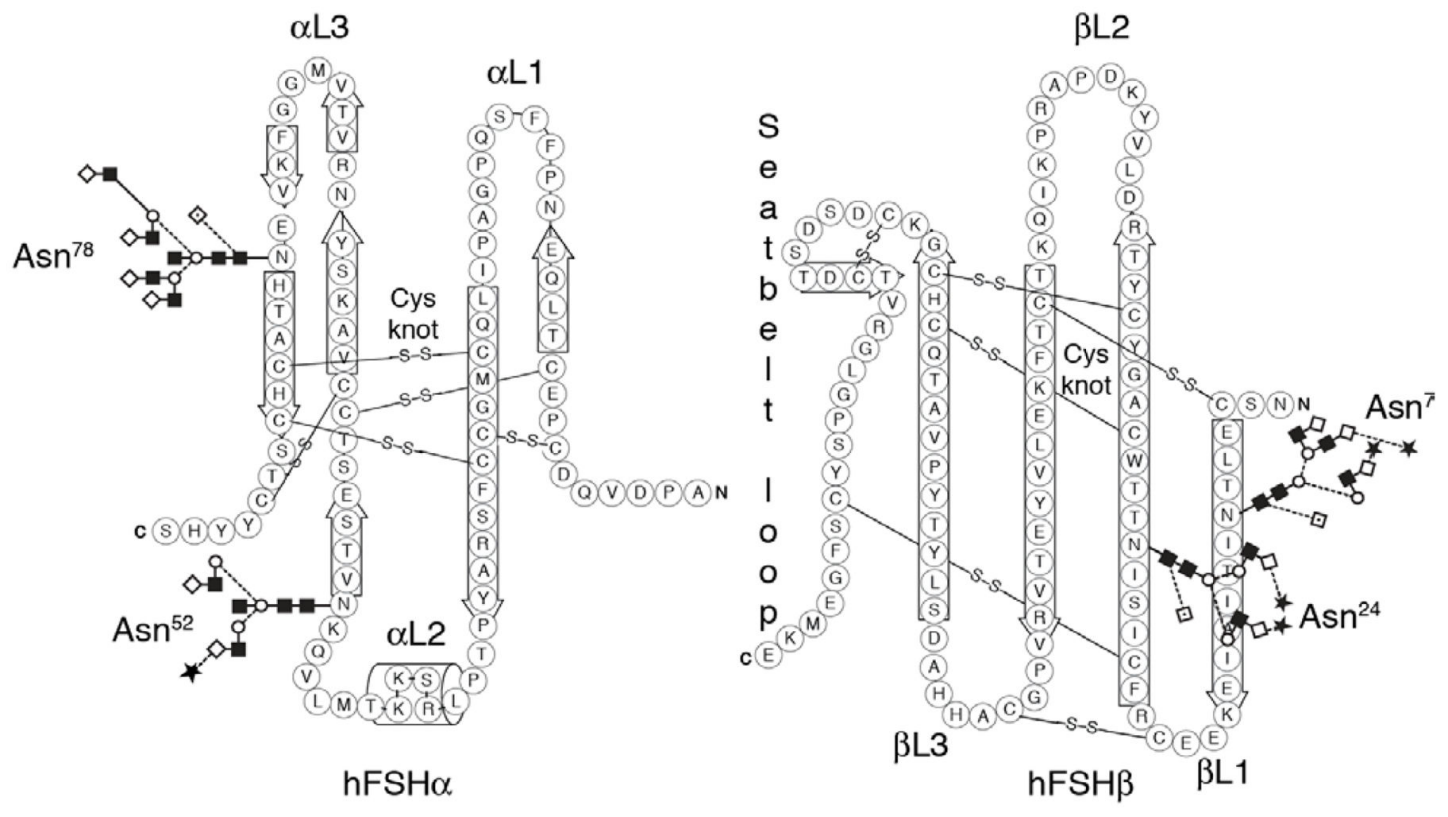
Cystine knot organization and glycosylation of human FSH α- and β-subunits. The cystine (Cys) knot disulfide bonds are indicated as lines. The loops are designated αL1, αL2, αL3, βL1, βL2, and βL3, as indicated. The FSHα seatbelt loop that embraces αL2 of FSHα in the heterodimer is indicated. The locations of the asparagine (Asn) N-glycosylation sites on loops αL2, αL2, and βL1 show diagrammatic representations of a glycan found at each site by glycopeptide mass spectrometry.

**Figure 2:**
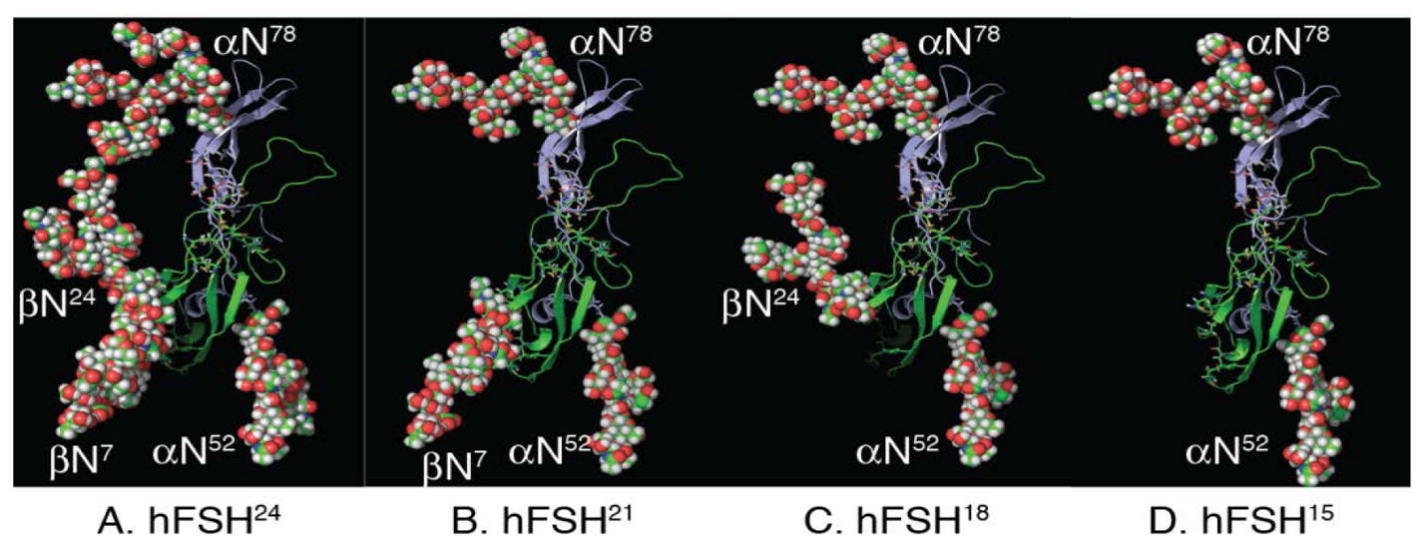
Human FSH glycoform models. The FSHα (green) and FSHβ (blue) subunits are shown as backbone cartoons. The N-glycans are shown as spheres and represent the most abundant glycans observed in glycopeptide mass spectra [6]. Panel A. hFSH^24^, which possesses all 4 N-glycans. Panel B. hFSH^21^, which lacks βAsn^24^ glycan. Panel C. hFSH^18^, which lacks βAsn^7^ glycan. Panel D. hFSH^15^, which lacks both FSHβ N-glycans. The hFSH^24^ model was created using Tripos Sybyl and subjected to molecular dynamics. The image in panel A was rendered with PyMol and the FSHβ glycans hidden in subsequent panels.
